# Mechanisms Underlying the Association of Chronic Obstructive Pulmonary Disease With Heart Failure

**DOI:** 10.1016/j.jcmg.2021.03.026

**Published:** 2021-10

**Authors:** Jakub Lagan, Erik B. Schelbert, Josephine H. Naish, Jørgen Vestbo, Christien Fortune, Joshua Bradley, John Belcher, Edward Hearne, Foluwakemi Ogunyemi, Richard Timoney, Daniel Prescott, Hamish D.C. Bain, Tasneem Bangi, Mahvash Zaman, Christopher Wong, Anthony Ashworth, Helen Thorpe, Robin Egdell, Jerome McIntosh, Bruce R. Irwin, David Clark, Graham Devereux, Jennifer K. Quint, Richard Barraclough, Matthias Schmitt, Christopher A. Miller

**Affiliations:** a Manchester University NHS Foundation Trust, Wythenshawe Hospital, Manchester, United Kingdom; b Division of Cardiovascular Sciences, School of Medical Sciences, Faculty of Biology, Medicine and Health, Manchester Academic Health Science Centre, University of Manchester, Manchester, United Kingdom; c Department of Medicine, University of Pittsburgh School of Medicine, Pittsburgh, Pennsylvania, USA; d Cardiovascular Magnetic Resonance Center, Heart and Vascular Institute, University of Pittsburgh Medical Center, Pittsburgh, Pennsylvania, USA; e Clinical and Translational Science Institute, University of Pittsburgh, Pittsburgh, Pennsylvania, USA; f Division of Infection, Immunity, and Respiratory Medicine, School of Biological Sciences, Faculty of Biology, Medicine and Health, Manchester Academic Health Science Centre, University of Manchester, Manchester, United Kingdom; g Stepping Hill Hospital, Stockport NHS Foundation Trust, Hazel Grove, Stockport, United Kingdom; h Royal Bolton Hospital, Bolton NHS Foundation Trust, Farnworth, Bolton, United Kingdom; i Royal Albert Edward Infirmary, Wrightington, Wigan and Leigh NHS Foundation Trust, Wigan, United Kingdom; j Tameside Hospital, Tameside and Glossop Integrated Care NHS Foundation Trust, Ashton-under-Lyne, United Kingdom; k Trafford General Hospital, Manchester University NHS Foundation Trust, Davyhulme, Manchester, United Kingdom; l Fairfield General Hospital, Pennine Acute Hospitals NHS Trust, Oldham, United Kingdom; m Macclesfield District General Hospital, East Cheshire NHS Trust, Macclesfield, Cheshire, United Kingdom; n Liverpool School of Tropical Medicine, Liverpool, United Kingdom; o National Heart and Lung Institute, Imperial College London, London, United Kingdom; p Division of Cell-Matrix Biology and Regenerative Medicine, Wellcome Centre for Cell-Matrix Research, School of Biology, Faculty of Biology, Medicine and Health, Manchester Academic Health Science Centre, University of Manchester, Manchester, United Kingdom

**Keywords:** cardiac magnetic resonance, chronic obstructive pulmonary disease, heart failure, mortality, myocardial fibrosis, myocardial inflammation, CI, confidence interval, COPD, chronic obstructive pulmonary disease, ECV, extracellular volume, FEV_1_, forced expiratory volume in 1 s, FVC, forced vital capacity, HF, heart failure, HR, hazard ratio, LV, left ventricular, CMR, cardiac magnetic resonance, USPIO, ultrasmall superparamagnetic particles of iron oxide

## Abstract

**Objectives:**

The purposes of this study were to determine why chronic obstructive pulmonary disease (COPD) is associated with heart failure (HF). Specific objectives included whether COPD is associated with myocardial fibrosis, whether myocardial fibrosis is associated with hospitalization for HF and death in COPD, and whether COPD and smoking are associated with myocardial inflammation.

**Background:**

COPD is associated with HF independent of shared risk factors. The underlying pathophysiological mechanism is unknown.

**Methods:**

A prospective, multicenter, longitudinal cohort study of 572 patients undergoing cardiac magnetic resonance (CMR), including 450 patients with COPD and 122 age- and sex-matched patients with a median: 726 days (interquartile range: 492 to 1,160 days) follow-up. Multivariate analysis was used to examine the relationship between COPD and myocardial fibrosis, measured using cardiac magnetic resonance (CMR). Cox regression analysis was used to examine the relationship between myocardial fibrosis and outcomes; the primary endpoint was composite of hospitalizations for HF or all-cause mortality; secondary endpoints included hospitalizations for HF and all-cause mortality. Fifteen patients with COPD, 15 current smokers, and 15 healthy volunteers underwent evaluation for myocardial inflammation, including ultrasmall superparamagnetic particles of iron oxide CMR.

**Results:**

COPD was independently associated with myocardial fibrosis (p < 0.001). Myocardial fibrosis was independently associated with the primary outcome (hazard ratio [HR]: 1.14; 95% confidence interval [CI]: 1.08 to 1.20; p < 0.001), hospitalization for HF (HR: 1.25 [95% CI: 1.14 to 1.36]); p < 0.001), and all-cause mortality. Myocardial fibrosis was associated with outcome measurements more strongly than any other variable. Acute and stable COPD were associated with myocardial inflammation.

**Conclusions:**

The associations between COPD, myocardial inflammation and myocardial fibrosis, and the independent prognostic value of myocardial fibrosis elucidate a potential pathophysiological link between COPD and HF.

Chronic obstructive pulmonary disease (COPD) is associated with left ventricular heart failure (HF), independent of shared risk factors, and HF independently contributes to mortality in patients with COPD (1).

The pathophysiological mechanisms underlying the association remain unclear. Myocardial fibrosis, driven by myocardial inflammation occurring as part of systemic inflammation, is a widely held hypothesis, but evidence for this is lacking, and other mechanisms have been proposed (2, 3, 4). Elucidating the underlying pathophysiology would enable improved risk stratification and development and evaluation of therapies targeting the pathophysiology.

This multicenter study aimed to determine why COPD is associated with HF. Specifically, it aimed to answer the following questions. 1) Is COPD associated with myocardial fibrosis? 2) Is myocardial fibrosis associated with hospitalization for HF and death in patients with COPD? 3) Are COPD and smoking associated with myocardial inflammation?

## Methods

The study was approved by an ethics committee at each site, and all participants provided written informed consent.

### Part 1. Investigation of the relationship between COPD and myocardial fibrosis, and the relationship between myocardial fibrosis and outcome in COPD

#### Study design and participants

A prospective, multicenter, longitudinal cohort study was conducted in order to: 1) evaluate the relationship between COPD and myocardial fibrosis; and 2) to determine whether myocardial fibrosis is associated with hospitalization for HF and death in patients with COPD.

Consecutive adult patients undergoing clinical cardiac magnetic resonance (CMR) at University of Pittsburgh Medical Centre (UPMC) between June 2010 and April 2015, and at Manchester University NHS Foundation Trust (MFT) between January 2015 and July 2018, were prospectively recruited. Consecutive patients with diagnoses of stable COPD, as determined by their medical record, were identified. Randomly selected age- and sex-matched patients without COPD were identified as a control population. Exclusion criteria included amyloidosis, hypertrophic cardiomyopathy, acute myocardial infarction, myocarditis, takotsubo cardiomyopathy, and complex congenital heart disease.

### Study procedures

Cardiac CMR was performed using 3 scanners (1.5-T Avanto model, Siemens, Munich, Germany; 1.5-T Espree model, Siemens; and 3-T Skyra model, Siemens) and included steady-state free precession cine imaging (standard long- and short-axis views), basal and mid-left ventricular (LV) short-axis T1 mapping (MOdified Look-Locker Inversion Recovery [MOLLI]) pre- and post-gadolinium-based contrast agent (0.15 to 0.20 mmol/kg gadoterate meglumine [Dotarem], Guerbet, Princeton, New Jersey) or 0.20 mmol/kg gadoteridol (Prohance, Bracco Diagnostics, Milan, Italy) and late gadolinium enhancement imaging. Hematocrit and estimated glomerular filtration rate (eGFR) were measured on the same day. Clinically available pulmonary function tests were recorded.

### Cardiac CMR analysis

Volumetric analysis was performed in accordance with current guidelines, with papillary muscles included in measurement of myocardial mass (5). Myocardial fibrosis was measured using the extracellular volume (ECV) technique using the middle one-third of the LV short-axis myocardium (6). Infarcted myocardium was excluded from the ECV measurement while foci of nonischemic enhancement were included, according to Society for Cardiovascular Magnetic Resonance recommendations (7). CMR analysis was performed blinded to outcome data but not blinded to the presence or absence of COPD.

### Study outcomes

Data were managed using Research Electronic Data Capture (REDCap) (8). Baseline comorbidity data were determined from primary and secondary care medical records.

The primary endpoint was a composite of first hospitalization for HF after cardiac CMR or all-cause mortality. The secondary endpoints were: 1) first hospitalization for HF after cardiac CMR; and 2) all-cause mortality. First hospitalization for HF was recorded from primary and secondary care medical records and determined independently by the clinical team responsible for the patient’s care. Mortality status for UPMC patients was ascertained by Social Security Death Index queries and verified against patients’ medical records. Mortality status for patients with MFT was ascertained from primary and secondary care medical records.

### Part 2. Investigation of the relationship between COPD and myocardial inflammation, and smoking and myocardial inflammation

#### Study design and participants

A prospective observational study was conducted in order to determine whether: 1) COPD; and 2) smoking are associated with myocardial inflammation.

Consecutive consenting patients with an acute respiratory exacerbation of COPD, defined in accordance with the Global Initiative for COPD (9), between October 2017 and November 2018, managed by the clinical respiratory team at MFT, were prospectively recruited. Patients were evaluated on 2 occasions: 1) during the acute exacerbation, within 3 weeks of the onset of symptoms of the exacerbation (“acute”); and 2) when stable, a minimum of 2 months after the exacerbation (“stable”).

Consecutive consenting current smokers with a smoking history of ≥10 pack-years and without a diagnosis of COPD or significant airflow obstruction (defined as the ratio of forced expiratory volume in 1 s [FEV_1_; l] to the forced vital capacity [FVC; l] >0.7) were prospectively recruited through poster advertisement. Age- and sex-matched healthy volunteers (no cardiovascular symptoms, no history of medical conditions, and normal electrocardiogram results) were recruited by poster advertisement for comparisons. Smokers and healthy volunteers underwent 1 assessment.

Exclusion criteria included contraindication to CMR; estimated glomerular filtration rate <50 ml/min/1.73 m^2^; iron overload; absolute erythrocytosis or polycythemia; hypotension; known HF; or previous myocardial infarction.

### Study procedures

Evaluation included laboratory measurements (blood count, renal function, liver function, C-reactive protein, high-sensitivity troponin I), spirometry and CMR. Briefly, the CMR protocol included parametric mapping, dynamic contrast-enhanced CMR, ultrasmall superparamagnetic particles of iron oxide (USPIO)-enhanced CMR and late gadolinium-enhanced CMR. CMR was performed before and at 48 and 73 h following an infusion of USPIO (ferumoxytol, AMAG Pharmaceuticals, Waltham, Massachusetts). Myocardial R2∗ behavior over time and R2∗:R1 ratio were measured. USPIO are phagocytosed by active cardiac macrophages and this protocol specifically identifies myocardial inflammation (10). Supplemental Appendix shows details of protocol and analysis.

### Statistical analysis

Part 1 was powered for a multivariate linear regression analysis aimed at determining the relationship between COPD and myocardial fibrosis, measured using ECV. A total of 465 patients, including 365 patients with COPD and 100 age- and sex-matched patients without COPD, provided the study with 90% power to detect an increase in R2 after including the COPD variable in the multivariate model of 1% or more, assuming an R2 value of the model prior to including COPD of 0.55. A higher number of patients with COPD than anticipated were identified, thus the number of patients without COPD included was increased proportionately. Participants without COPD were selected using proportional random sampling with subgroups defined by 10-year age bands and sex from both sites separately to ensure that the proportions of COPD to age- and sex-matched patients without COPD from each site were consistent.

For Part 2, 15 patients with COPD were required to detect an absolute minimum difference between acute and stable scans, of 3 in terms of changes in R2∗:R1 ratio, with 80% power at a 5% significance level (2-sided), assuming a SD of the within-patient differences equal to 4.

Data were summarized using mean ± SD or median (interquartile range [IQR]) and were compared using Student’s *t*-tests or non-parametric equivalents as appropriate. Chi-squared test results were used to compare categorical variables. Linear regression models (univariate and stepwise multivariate) were used to assess the relationship among variables, including COPD, and myocardial fibrosis, measured using ECV. Cox regression analyses (univariate and backward stepwise multivariate with a p value of 0.05 for entry and 0.10 for removal) were used to evaluate the relationships between myocardial fibrosis, measured using ECV, and each of the outcomes in patients with COPD, stratified by site. For the time to hospitalizations for HF analyses, dying patients were censored at the time point of their death. For consistency between sites, duration of follow-up for UPMC patients was censored at 1,537 days, which was the longest follow-up duration for MFT patients. The models satisfied the global Schoenfeld test for proportional hazards. The plots of the individual covariate scaled Schoenfeld residuals against time also demonstrated no evidence of a relationship with time (Supplemental Figure 1). The p value for ECV was borderline (0.05); however, it was preferable to fit a common time effect for ECV for simplicity, and tests of continuous covariate linearity further showed the inclusion of unadjusted ECV. ECV did not interact with the other variables used in the regression models. Wald tests for model coefficients using the chi-squared distribution were used as an indication of the strength of relationships between variables and outcome. Kaplan-Meier curves used the log-rank test with ECV categorized below and above the median. Generalized estimating equations were used to compare USPIO-related change in magnetic relaxation rates over time (10). Briefly, analyses were conducted using a robust estimator covariance matrix and an exchangeable working correlation matrix. A hybrid parameter estimation method was used with maximum-likelihood estimate for the scale parameter method. Correlation analysis was performed using Pearson or Spearman test correlation as appropriate. Statistical analyses were performed using SPSS version 22 software (IBM, Armonk, New York).

## Results

### Relationship between COPD and myocardial fibrosis

The cohort consisted of 572 patients, including 450 patients with COPD and 122 randomly selected age- and sex-matched patients without COPD. Baseline characteristics are summarized in Table 1, Supplemental Results, Supplemental Tables 1 and 2. Pulmonary function tests were available in 235 patients with COPD. Mean FEV_1_ was 67.6% ± 21.1%. COPD was associated with a higher burden of myocardial fibrosis (median ECV: 28.0% [IQR: 25.8% to 31.1%] vs. 26.1% [IQR: 23.7% to 28.8%; p < 0.001). Current smoking, ever smoking (defined as current or previous smoking), and diabetes mellitus were more common in patients with COPD. COPD was associated with lower LV ejection fraction (55% [44% to 65%] vs. 60% [51% to 70%], respectively; p < 0.001) and RV ejection fraction (56% [49% to 63%] vs. 62% [55% to 66%], respectively; p < 0.001).

**Table 1:** Participant Characteristics

	COPD (n = 450)	Non–COPD (n = 122)	p Value
Demographics			
Age, yrs	65 (58–72)	64 (57–72)	0.819
Males	290 ± 64	79 ± 65	1.000
Scanning details			
Hospital MFT:UPMC	363:87 (81:19)	100:22 (82:18)	0.796
Scanner 1.5-T: 3-T	258:192 (57:43)	68:54 (56:44)	0.758
Comorbidities			
Diabetes mellitus	111 ± 25	17 ± 14	0.014
Hypertension	236 ± 52	52 ± 42	0.066
Dyslipidemia	249 ± 55	71 ± 58	0.608
Atrial fibrillation	84 ± 19	25 ± 21	0.697
Coronary revascularizations	111 ± 25	34 ± 28	0.483
Current smoker	133 ± 30	13 ± 11	<0.001
Ever smoker	391 ± 87	65 ± 53	<0.001
BSA, m^2^	1.9 (1.7–2.1)	2.0 (1.8–2.2)	0.038
Laboratory and CMR findings			
LV EDV/BSA, ml/m^2^	81 (64–102)^a^	84 (73–96)	0.093
LV ESV/BSA, ml/m^2^	35 (24–56)^a^	32 (24–45)	0.248
LVEF %	55 (44–65)^a^	60 (51–70)	<0.001
LV mass/BSA, g/m^2^	63 (51–77)^a^	65 (53–75)	0.853
MI present	154 ± 34^b^	44 ± 36	1.000
RV EDV/BSA, ml/m^2^	39 (32–47)^e^	40 (35–47)^f^	0.252
RV ESV/BSA, ml/m^2^	17 (13–23)^e^	15 (13–19)^f^	0.227
RVEF %	56 (49–63)^e^	62 (55–66)^f^	<0.001
ECV %	28.0 (25.8–31.1)^c^	26.1 (23.7–28.8)	<0.001
eGFR, ml/min/1.73 m^2^	78 (62–90)^d^	79 (68–90)	0.633
Hematocrit %	41.3 (37.9–44.9)^b^	41.3 (39.2–44.4)	0.614

Values are median (interquartile range) or mean ± SD depending on distribution.

BSA = body surface area; CMR = cardiac magnetic resonance; COPD = chronic obstructive pulmonary disease; ECV = extracellular volume; EDV = end diastolic volume; EF = ejection fraction; eGFR = estimated glomerular filtration rate; ESV = end systolic volume; LV = left ventricle; MFT = Manchester University NHS Foundation Trust; MI = myocardial infarction; UPMC = University of Pittsburgh Medical Centre.

a n = 435;

b n = 425;

c n = 393 (it was not possible to calculate ECV in 57 patients because the native or post–contrast T1 maps were not acquired or because same–day hematocrit was not available);

d n = 449;

e n = 348;

f n = 100.

Univariate and multivariate associations with myocardial fibrosis are displayed in Table 2. In multivariate analysis, COPD was independently associated with myocardial fibrosis (unstandardized coefficient B: 1.15; SE: 0.35; p = 0.001).

**Table 2:** Univariate and Multivariate Associations With Myocardial Fibrosis, Measured Using Myocardial Extracellular Volume

	Univariate Model (n = 515^b^)			Multivariate Model (n = 514)		
	t Value	B ± SE	p Value	t Value	B ± SE	p Value
Demographics						
Age (per 1 yr increase)	0.28	0.01 ± 0.02	0.784			
Males	−3.72	−1.40 ± 0.38	<0.001			
BSA (per 0.01 m^2^ increase)	−5.50	−0.04 ± 0.66	<0.001	−3.83	−0.02 ± 0.01	<0.001
UPMC hospital	5.82	2.53 ± 0.44	<0.001			
Comorbidities						
COPD	4.91	2.07 ± 0.42	<0.001	3.25	1.15 ± 0.35	0.001
Current smoker	5.00	2.06 ± 0.41	<0.001	3.33	1.17 ± 0.35	0.001
Ever smoker	3.81	1.71 ± 0.45	<0.001			
Diabetes mellitus	1.18	0.51 ± 0.43	0.240			
Hypertension	0.56	0.21 ± 0.37	0.574			
Dyslipidemia	−2.98	−1.09 ± 0.37	0.003			
Atrial fibrillation	−1.08	−0.50 ± 0.46	0.282			
Coronary revascularizations	−3.73	−1.57 ± 0.42	<0.001	−3.79	−1.30 ± 0.34	<0.001
Laboratory and CMR findings						
LVEF (per 1% increase)	−6.49	−0.07 ± 0.01	<0.001	−7.49	−0.07 ± 0.01	<0.001
LV mass (per 1 g/m^2^ increase)	3.08	0.03 ± 0.01	0.002			
MI present	−0.62	−0.24 ± 0.38	0.538			
eGFR^a^	1.34	0.01 ± 0.01	0.182	2.06	0.02 ± 0.01	0.040
Hematocrit %	−11.23	−0.34 ± 0.03	<0.001	−12.49	−0.35 ± 0.03	<0.001

Values are mean ± SD depending on distribution.

CMR = cardiac magnetic resonance; other abbreviations as in Table 1.

a n = 514, per 1 ml/min per 1.73 m^2^ increase.

b n = 515 consisting of 393 patients with COPD and 122 non–COPD patients, as shown in Table 1.

### Relationship between myocardial fibrosis and outcome in COPD

The cohort consisted of the same 450 patients with COPD. During a median follow-up period of 726 days (interquartile range: 492 to 1,160 days), 101 patients were either hospitalized for HF or died. Thirty-six patients were hospitalized for HF, and 77 patients died. No patient was lost to follow-up.

**Figure 1:**
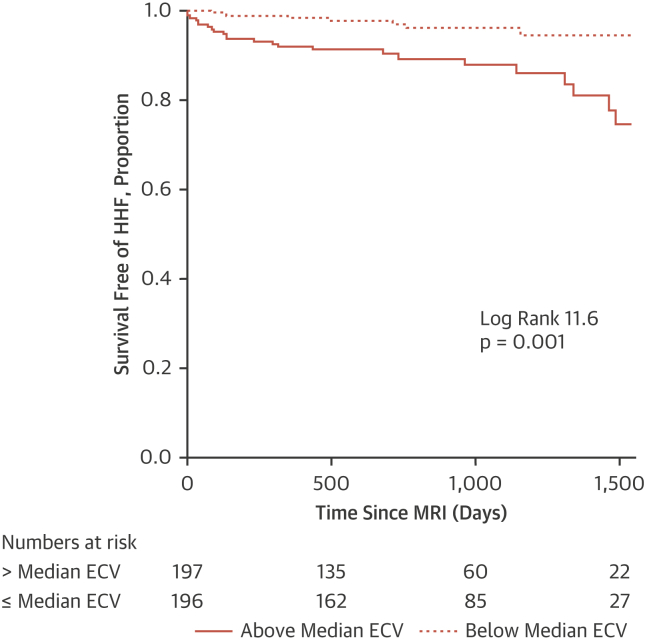
Myocardial Fibrosis and Survival Free From Hospitalization for Heart Failure Kaplan-Meier curve for survival free from hospitalization for heart failure in patients with chronic obstructive pulmonary disease, according to myocardial fibrosis burden. Myocardial fibrosis was measured using cardiac magnetic resonance extracellular volume (ECV). MRI = magnetic resonance imaging.

In multivariate Cox regression analysis, myocardial fibrosis was independently associated with the composite primary outcome of hospitalization for HF or all-cause mortality (hazard ratio [HR] per 1% increase in ECV: 1.14; 95% confidence interval (CI): 1.08 to 1.20; p < 0.001) (Table 3, Central Illustration), hospitalizations for HF (HR: 1.25; 95% CI: 1.14 to 1.36; p < 0.001) (Table 3, Figure 1), and all-cause mortality (HR: 1.13; 95% CI: 1.06 to 1.19; p < 0.001) (Supplemental Table 3, Supplemental Figure 2). Myocardial fibrosis was more strongly associated with each of the outcomes than any other variable. Substituting ECV for native myocardial T1 (longitudinal relaxation time) or post-contrast myocardial T1 revealed that neither were associated with the primary outcome (Supplemental Tables 4 and 5).

**Table 3:** Cox Regression Modeling of the Combined Endpoint (Hospitalization for Heart Failure or All-Cause Mortality) and of Hospitalization for Heart Failure Alone

	Combined Endpoint						Hospitalization for Heart Failure					
	Univariate Model (n = 450)			Multivariate model (n = 392)^e^			Univariate Model (n = 450)			Multivariate Model (n = 392)^e^		
	Chi-Square Test	HR (95% CI)	p Value	Chi-Square Test	HR (95% CI)	p Value	Chi-Square Test	HR (95% CI)	p Value	Chi-Square Test	HR (95% CI)	p Value
Demographics												
Age (per 1 yr increase)	0.46	1.01 (0.99 to 1.03)	0.499				0.05	1.00 (0.97–1.03)	0.819			
Male	1.59	1.31 (0.86 to 2.00)	0.207				0.20	0.86 (0.44 to 1.03)	0.655	5.49	0.34 (0.14 to 0.84)	0.019
BSA (per 0.01 m^2^ increase)	1.12	1.00 (1.00 to 1.01)	0.289				2.00	1.01 (1.00 to 1.02)	0.157	9.17	1.02 (1.01 to 1.04)	0.002
Comorbidities												
Current smoker	0.72	1.20 (0.79 to 1.80)	0.395				0.02	1.05 (0.52 to 2.11)	0.896			
Ever smoker	0.76	1.32 (0.71 to 2.47)	0.384				0.01	0.97 (0.38 to 2.49)	0.944			
Diabetes mellitus	3.74	1.50 (1.00 to 2.27)	0.053				4.85	2.11 (1.09 to 4.09)	0.028	5.08	2.33 (1.12 to 4.86)	0.024
Hypertension	3.06	1.44 (0.96 to 2.15)	0.080				2.20	1.69 (0.84 to 3.40)	0.138			
Dyslipidemia	2.94	0.71 (0.48 to 1.05)	0.086				0.19	0.87 (0.45 to 1.67)	0.667			
Atrial fibrillation	1.41	1.33 (0.83 to 2.14)	0.236				0.01	0.96 (0.40 to 2.30)	0.922			
Coronary revascularizations	3.52	1.49 (0.98 to 2.27)	0.061	10.24	2.13 (1.34 to 3.38)	0.001	0.04	0.92 (0.42 to 2.02)	0.838			
Laboratory and CMR findings												
LV EF (per 1% increase)^a^	10.26	0.98 (0.97 to 0.99)	0.001				11.95	0.97 (0.95 to 0.99)	0.001			
LV mass (per 1 g/m^2^ increase)^a^	6.71	1.01 (1.00 to 1.02)	0.010	7.67	1.01 (1.00 to 1.02)	0.006	14.42	1.02 (1.01 to 1.03)	<0.001	14.82	1.03 (1.01–1.04)	<0.001
MI present^b^	5.27	1.60 (1.07 to 2.39)	0.022				1.41	1.51 (0.77 to 2.97)	0.235			
ECV (per 1% increase)^c^	30.45	1.15 (1.09 to 1.21)	<0.001	23.76	1.14 (1.08 to 1.20)	<0.001	27.99	1.23 (1.14 to 1.33)	<0.001	24.88	1.25 (1.14 to 1.36)	<0.001
eGFR^d^	3.65	0.99 (0.98 to 1.00)	0.056				1.93	0.99 (0.97 to 1.01)	0.164			
Hematocrit (per 1% increase)^b^	18.42	0.93 (0.90 to 0.96)	<0.001				9.31	0.92 (0.87 to 0.97)	0.002			

CI = confidence interval; CMR = cardiac magnetic resonance; HR = hazard ratio; other abbreviations as in Tables 1 and 2.

a n = 435;

b n = 425;

c n = 393;

d n = 449, per 1 ml/min per 1.73 m^2^ increase;

e stepwise model was performed in 392 patients with complete data.

**Central Illustration:**
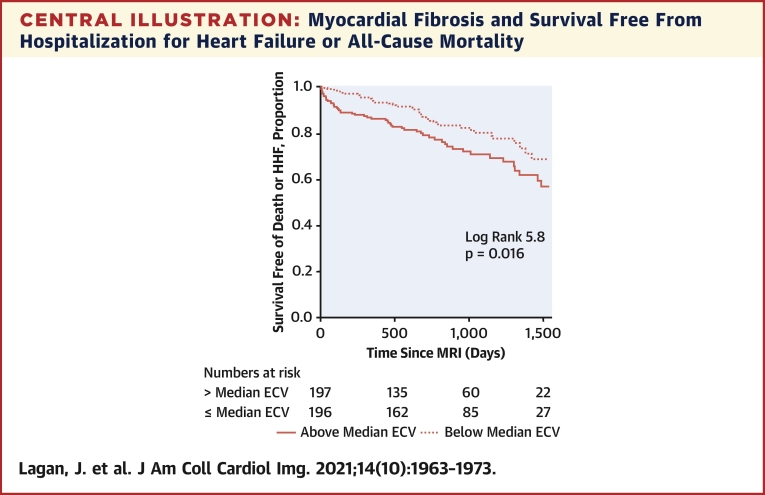
Myocardial Fibrosis and Survival Free From Hospitalization for Heart Failure or All-Cause Mortality Kaplan-Meier curve for survival free from a composite of all-cause mortality or hospitalization for heart failure (HHF) in patients with chronic obstructive pulmonary disease, according to myocardial fibrosis burden. Myocardial fibrosis was measured using cardiac magnetic resonance extracellular volume (ECV). MRI = magnetic resonance imaging.

When FEV_1_ was included in the multivariate Cox regression model, myocardial fibrosis was the only variable independently associated with the composite outcome of hospitalization for HF or all-cause mortality (HR: 1.15; 95% CI: 1.06 to 1.25; p < 0.001) (Supplemental Table 6). Similarly, myocardial fibrosis remained independently associated with the composite outcome when RV ejection fraction was included in the model (Supplemental Table 7).

### Relationship between COPD and myocardial inflammation

The study consisted of 15 patients with an acute respiratory exacerbation of COPD. Patient characteristics are summarized in Supplemental Table 8. The acute evaluation was performed at a median of 16 days (15 to 20 days) after exacerbation onset. Ten patients returned for the stable evaluation, which was performed at a median of 119 days (102 to 140 days) after exacerbation onset. The reasons for not returning for the stable evaluation were: claustrophobia (n = 2), breathlessness (n = 1), mild reaction to USPIO (n = 1), and clinical deterioration (n = 1). According to an FEV_1_ measurement performed at the stable evaluation, 1 patient (10%) had mild COPD, 7 (70%) had moderate COPD, and 2 (20%) had severe COPD. Fifteen healthy volunteers were recruited (Supplemental Table 8).

Acute COPD was associated with elevated C-reactive protein and white cell counts, which were lower in patients with stable COPD but did not return to normal (Supplemental Table 9). Two patients demonstrated evidence of chronic myocardial infarction on late enhanced imaging that was otherwise unrecognized clinically. Infarcted myocardium was excluded from all parametric analyses.

**Figure 2:**
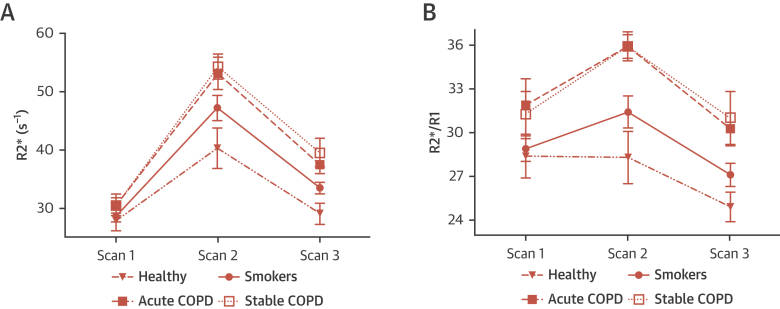
USPIO-Enhanced CMR Measurements in Patients With COPD During Acute Exacerbation (Acute COPD) and When Stable (Stable COPD), Current Smokers, and Healthy Volunteers **(A)** Myocardial R2∗. **(B)** Myocardial R2∗/R1 ratio. Scan 1 = baseline CMR scan before USPIO administration. Scans 2 and scan 3 = CMR scans performed at 48 and 72 hours, respectively, following USPIO administration. CMR = cardiac magnetic resonance; COPD = chronic obstructive pulmonary disease; USPIO = ultrasmall superparamagnetic particles of iron oxide.

USPIO-CMR demonstrated evidence of myocardial inflammation in COPD at both the acute and stable time points (Supplemental Tables 9 and 10, Figure 2). Myocardial R2∗ behavior over time (p = 0.038) and R2∗:R1 ratio (p = 0.005) were significantly higher in acute COPD patients than in healthy volunteers, and in stable patients with COPD than in healthy volunteers (p = 0.005 and p = 0.025, respectively). There were no significant differences between acute and stable COPD. Indicators of myocardial edema were inconsistent; there were no differences in myocardial T2 relaxation time between acute and stable COPD, although myocardial T1 was higher during the acute than during the stable period. Myocardial capillary permeability was numerically higher during acute COPD, but the differences were not statistically significant.

### Relationship between smoking and myocardial inflammation

The study consisted of 15 current smokers. Participant characteristics are summarized in Supplemental Table 8. Smokers demonstrated intermediate USPIO-CMR measurements that were between those from healthy volunteers and those with COPD (Figure 2). Myocardial R2∗ behavior over time (p = 0.015) (Supplemental Table 10) was significantly higher in smokers than in healthy volunteers but differences in R2∗:R1 ratios were not statistically significant (Supplemental Table 9, Figure 2). There was no evidence of myocardial edema or increased capillary permeability.

There were significant relationships between the severity of myocardial inflammation and biochemical evidence of myocardial injury (correlation of myocardial R2∗:R1 ratio with circulating high-sensitivity troponin I; r = 0.56; p = 0.004) and severity of airflow limitation (correlation of myocardial R2∗:R1 ratio with FEV_1_ r = −0.50; p = 0.010) in stable COPD and smokers.

## Discussion

This study found COPD was associated with myocardial fibrosis, independent of demographics, comorbidities, and other cardiac structural and functional factors. Furthermore, in patients with COPD, myocardial fibrosis was independently predictive of hospitalization for HF and death. The study also showed COPD to be associated with myocardial inflammation. These findings provide, for the first time, potential evidence for a pathophysiological mechanism underlying the association between COPD and HF.

The reported prevalence of HF in patients with COPD ranges between approximately 7% and 31%, but the broad “type” of HF with which COPD associates (i.e., reduced vs. preserved ejection fraction [EF]) has been poorly characterized (11). In the current study, COPD was associated with lower LVEF than matched patients without COPD, but in most of the patients, LVEF was within the accepted normal range or at most mildly reduced, and LVEF was not independently associated with hospitalization for HF or death, thus suggesting that the predominant type of HF associated with COPD is HF with preserved EF.

Although large epidemiological studies have demonstrated that the association between COPD and HF is independent of common risk factors such as ischemic heart disease, diabetes, and hypertension, the mechanism responsible for the association has remained unclear (1). Myocardial fibrosis, driven by myocardial inflammation occurring as part of systemic inflammation leading to mechanical, electric, and vasomotor dysfunction of the myocardium is a widely held hypothesis (2). Indirect evidence for the hypothesis includes the association of circulating inflammatory and fibrotic biomarkers with LV diastolic dysfunction and incident HF and with airflow limitation in patients with COPD, but there has been no direct evidence (3,12, 13, 14, 15). Other mechanisms, such as reduced LV preload secondary to pulmonary dysfunction, increased afterload due to arterial stiffness, and autonomic dysfunction, have also been proposed (3,4).

Post mortem studies and a small previous cardiac CMR study have previously demonstrated evidence of myocardial fibrosis in COPD (16,17). However, those studies were confounded by comorbidities that are frequent in patients with COPD and are themselves associated with myocardial fibrosis. The large sample size of the current study means that, for the first time, COPD per se has been shown to be independently associated with myocardial fibrosis. Crucially, this study also shows that myocardial fibrosis is independently associated with hospitalization for HF and death in patients with COPD. Indeed, myocardial fibrosis was the strongest predictor of adverse outcome, outperforming comorbidities, traditional cardiac indices, and the key spirometry COPD index, FEV_1_.

These novel findings have important implications for the clinical management of patients with COPD and future research. Risk stratification in cases of COPD is imprecise, and prognostic models are poorly discriminative (18). The unparalleled risk stratification provided by the burden of myocardial fibrosis in the current study means that this measurement could be considered in the prognostication of patients with COPD. Furthermore, trials of therapeutics that target myocardial fibrosis, with the aim of improving the outcome of patients with COPD, are warranted.

Elevated circulating troponin levels are observed in acutely exacerbating and chronic stable COPD, the magnitude of which in the latter is associated with the severity of airflow obstruction and circulating markers of immune activation (13,19). Myocardial inflammation-induced cardiomyocyte injury has been proposed as a possible mechanism, but evidence for this has been lacking. The current study demonstrates, for the first time, that both acutely exacerbating and stable COPD are associated with myocardial inflammation in the form of myocardial macrophage infiltration. Indeed, in keeping with the aforementioned circulating biomarker studies, the magnitude of myocardial inflammation in the current study was associated with severity of airflow obstruction and circulating troponin levels. USPIO-CMR was used due to its specificity for myocardial inflammation. Circulating inflammatory biomarkers are not specific to the myocardium and standard cardiac CMR techniques and fluorine-18-labeled fluorodeoxyglucose positron emission tomography are not specific for inflammation.

Acute exacerbation of COPD is associated with greater systemic inflammation than stable COPD, and circulating troponin levels are generally higher during acute exacerbation, although comparative troponin data are limited, and the differences may be due to nebulized beta-_2_-agonist-induced tachycardia in the acute phase (13,20,21). In the current study, the severity of myocardial inflammation observed during acute exacerbation was similar to that in the stable period. More severe myocardial inflammation may have been evident if the CMR scan had been undertaken closer to the onset of the exacerbation (the interval was 16 days), but patient breathlessness prevented it from being performed earlier (Supplemental Appendix).

Although the present study elucidates a potential pathophysiological mechanism underlying the association between COPD and HF, it does not establish causality, and further studies are required.

### Study limitations

Participants in Part 1 underwent clinical cardiac CMR scanning; thus, they might not be representative of a more general COPD population. Spirometric data were not available for 48% of patients with COPD, thus it is possible that, despite their clinical diagnoses, some patients might not have had COPD. Nevertheless, despite this potential null bias that might have reduced the likelihood of detecting associations, significant associations were identified. CMR analysis was performed blinded to outcome data but was not blinded to the presence or absence of COPD, which could introduce bias. Reasons for hospitalization were determined by the clinical team responsible for patients’ care. Differentiating between hospitalization due to HF and hospitalization due to COPD exacerbation can be challenging, but with the full clinical information available, the clinical teams are well placed to make this differentiation (22), and it is the clinical team that determines how the patient is managed. Furthermore, the clinical team was independent of the research team. Patients were scanned using 3 scanners, at 2 magnetic field strengths, in 2 centers, which could introduce variability. Nevertheless, the association between ECV and outcome was demonstrated despite this. Although the relationship between ECV and histological myocardial fibrosis has been demonstrated in a number of previous studies, histological validation was not performed in the current study, and the relationship between ECV and histological myocardial fibrosis demonstrated in the previous studies may not necessarily extrapolate to the current study (6,7). In Part 2, patients with COPD were older than current smokers; thus, it was not possible to simultaneously match ages of healthy volunteers to both groups. However, the impact of age on myocardial T2∗ and T1 is minimal, and patients with COPD served as their own controls for the acute and stable comparisons (23, 24, 25, 26, 27). Sample size was smaller than in Part 1, although important between-group differences were identified. The possible impact of the timing of the acute CMR is discussed above. USPIO are phagocytosed by active cardiac macrophages, which are widely considered to be part of tissue inflammatory response, but they are not specific to the initiating injury. Thus, this study is unable to determine the cause of the inflammatory response.

## Conclusions

The associations among COPD, myocardial inflammation and myocardial fibrosis, and the independent prognostic value of myocardial fibrosis elucidate a potential pathophysiological link between COPD and HF. The findings have the potential to improve patient risk stratification and to be the basis for developing new therapeutic strategies for patients with COPD.

Perspectives **COMPETENCY IN MEDICAL KNOWLEDGE:** The associations among COPD, myocardial inflammation, and myocardial fibrosis, and the independent prognostic value of myocardial fibrosis, elucidate a potential pathophysiological link between COPD and heart failure.

**TRANSLATIONAL OUTLOOK:** The findings have the potential to improve patient risk stratification and be the basis for developing new therapeutic strategies for patients with COPD.

## Funding Support and Author Disclosures

The study was supported by a research grant from Guerbet. Guerbet had no role in the design and conduct of the study; collection, management, analysis, and interpretation of the data; preparation or approval of the manuscript; and the decision to submit the manuscript for publication. Dr. Lagan was funded by a Clinical Research Training Fellowship from the British Heart Foundation (FS/17/47/32805). Dr. Miller is funded by a Clinician Scientist Award (CS-2015-15-003) from the National Institute for Health Research. The views expressed in this publication are those of the authors and not necessarily those of the NHS, the National Institute for Health Research or the Department of Health. Dr. Vestbo is supported by the National Institute for Health Research Manchester Biomedical Research Centre. The work was also supported in part by a British Heart Foundation Accelerator award to The University of Manchester (AA/18/4/34221). The authors have reported that they have no relationships relevant to the contents of this paper to disclose.
