# Supplementary material for: Mechanisms Underlying the Association of Chronic Obstructive Pulmonary Disease With Heart Failure
Source: JACC Cardiovasc Imaging. 2021 Oct;14(10):1963–73. doi: 10.1016/j.jcmg.2021.03.026 (PMC8490158; doi:10.1016/j.jcmg.2021.03.026)
Supplement: Supplemental Data [file mmc1.docx]

**The association of chronic obstructive pulmonary disease with heart failure**

**Supplementary material**

**Supplementary methods**

**Part 2. Investigation of the relationship between COPD and myocardial inflammation, and smoking and myocardial inflammation**

**Magnetic resonance imaging (MRI) procedure**

MRI was performed at 1.5 T (Avanto, Siemens Medical Imaging). Baseline MRI scanning included steady-state free precession (SSFP) cine imaging (standard long- and short-axis views), basal and mid left ventricular (LV) T2* mapping (black blood multi-gradient-echo sequence; eight echo times, range 2.59-18.2ms), T1 mapping (MOdified Look-Locker Inversion Recovery (MOLLI)) and T2 mapping (T2-prepared SFFP), dynamic contrast enhanced (DCE) imaging using free-breathing 2D saturation-recovery fast low angle shot dynamic acquisitions and late gadolinium enhancement (LGE) imaging. Gadolinium-based contrast agent (gadoterate meglumine [Dotarem], Guerbet, France; 3ml/s) administration was divided into three boluses: an initial low dose (0.005 mmol/kg) that was followed by 2 minutes of dynamic acquisition, a second higher dose (0.05 mmol/kg) that was followed by 6 minutes of dynamic acquisition, and a final dose (0.1 mmol/kg).

Following the baseline MRI, an intravenous infusion of ultrasmall superparamagnetic particles of iron oxide (USPIO; ferumoxytol, AMAG Pharmaceuticals, USA) at a dose of 4mg/kg diluted in 0.9% saline, was administered over 30 minutes and followed by 30 minutes of observation.

Two MRI scans were performed at a median of 48h (47-49h) and 73h (71-74h) post-USPIO administration. There were no differences in scan timing between groups. Scanning included SSFP cine imaging, T2* and T1 mapping at positions corresponding to those acquired during the baseline scan.

**MRI analysis**

Volumetric analysis was performed using CVI42 (Circle Cardiovascular Imaging, Canada) in accordance with current guidelines.(1) T1 and T2 maps, to provide assessment of myocardial oedema, were generated in Siemens Argus (Siemens Medical Imaging) and transferred into Horos (Horos2K v2.2.0, The Horos Project) where epicardial, endocardial and blood pool regions of interest (ROI) were drawn. DCE imaging was analysed using custom written Matlab code (v9.0, The MathWorks, USA). Cardiac motion correction was achieved using an intensity-based rigid registration algorithm. Contrast agent kinetics were modelled using the extended version of the Kety model on a voxel-by-voxel basis within registered ROIs to calculate the transfer constant, Ktrans (2). Ktrans describes transendothelial transport of contrast medium (3) and reflects capillary permeability. Vascular permeability, and hence Ktrans, increases as a consequence of inflammatory processes (4). For the USPIO-MRI analysis, T2* maps were generated in MatLab and, together with T1 maps, were transferred into Horos. ROIs were drawn as described above. Weighted mean voxel R1 (longitudinal relaxation rate; 1/T1) and R2* (transverse relaxation rate in the presence of static field inhomogeneities; 1/T2*) were calculated. R2* behaviour over time and R2*/R1 ratio on the second scan post-USPIO were used to assess for active myocardial macrophage USPIO accumulation whilst taking account of passive tissue wash-through. We have previously provided histological validation of the USPIO technique, confirming the utility of USPIO for identifying active cardiac macrophages, and demonstrated that the described multiparametric, multi-time point MRI protocol differentiates active USPIO uptake from passive tissue wash through.(5)

USPIO infusion was generally well tolerated. One patient developed mild bronchospasm which resolved quickly after stopping the USPIO infusion and administering nebulised bronchodilators. This patient did not undergo subsequent MRI.

**Supplementary results**

**Part 1. Spirometry data**

Spirometry data was available for 235 patients with COPD. COPD severity: mild (n=67; 28%), moderate (n=117, 50%), severe (n=45, 19%), very severe (n=6, 3%) (6). There was a weak negative correlation between ECV and FEV1 (r= -0.181; p=0.011).

**Part 1. Extracellular volume (ECV) data**

There was no difference in ECV measured on the 1.5T compared to the 3T scanner at Manchester University NHS Foundation Trust (27.4% (25.0 – 30.4%) vs (26.8% (24.4 – 29.2%); p=0.125). ECV was higher at University of Pittsburgh Medical Centre (all 1.5T) compared to Manchester University NHS Foundation Trust (29.6% (26.9 – 32.7%); p<0.01). In order to account for this, Cox regression analyses were stratified by site. A breakdown of native T1, post-contrast T1 and ECV can be found in Supplementary Table 2. Categorising ECV into tertiles demonstrated a ‘dose-response’ relationship between ECV and outcome (See Supplementary Figure 3).

**Part 2. Relationship between COPD and myocardial inflammation**

There was a non-significant decrease in circulating C reactive protein (CRP) and white cell count (WCC) between the clinical assessment at the beginning of the exacerbation and the Acute evaluation (CRP: 8mg/L [interquartile range (IQR) 2–22mg/L] to 6mg/L [3–11mg/L]; p=0.07. WCC: 10.9x10^9^/L [7.7–13.5x10^9^/L] to 9.6x10^9^/L [8.0– 11.0x10^9^/L]; p=0.102). Three patients were unable to complete the baseline MRI due to breathlessness (one patient) and claustrophobia (two patients).

Lung function tests in the stable period were as follows: forced expiratory volume in one second (FEV_1_) 1.3±0.4L/s; percentage of predicted FEV_1_ 54.3±15.6%; forced vital capacity (FVC) 2.1±0.7L; percentage of predicted FVC 70.4±20.7%; FEV1/FVC ratio 63.5±13.3%.

**Part 2. Relationship between smoking and myocardial inflammation**

Lung function tests in smokers were as follows: FEV_1_ 3.0±1.0L/s; percentage of predicted FEV_1_ 89.8±16.4%; FVC 3.8±1.2L; percentage of predicted FVC 93.8±16.6%; FEV1/FVC ratio 78.2±5.6%.

**References:**

1. Schulz-Menger J, Bluemke DA, Bremerich J et al. Standardized image interpretation and post processing in cardiovascular magnetic resonance: Society for Cardiovascular Magnetic Resonance (SCMR) board of trustees task force on standardized post processing. J Cardiovasc Magn Reson 2013;15:35.

2. Naish JH, Kershaw LE, Buckley DL, Jackson A, Waterton JC, Parker GJM. Modeling of contrast agent kinetics in the lung using T1-weighted dynamic contrast-enhanced MRI. Magn Reson Med 2009;61:1507-1514.

3. Leach MO, Brindle KM, Evelhoch JL et al. The assessment of antiangiogenic and antivascular therapies in early-stage clinical trials using magnetic resonance imaging: issues and recommendations. Br J Cancer 2005;92:1599-1610.

4. Claesson-Welsh L. Vascular permeability--the essentials. Ups J Med Sci 2015;120:135-143.

5. Lagan J, Naish JH, Simpson K et al. Substrate for the myocardial inflammation–heart failure hypothesis identified using novel USPIO methodology. JACC Cardiovasc Imaging 2020 Apr 15 [Epub ahead of print].

6. Global Initiative for the Diagnosis Management and Prevention of Chronic Obstructive Pulmonary Disease. Gold Report. 2019.

**Supplementary Figure 1. Example Schoenfeld plots.**

**
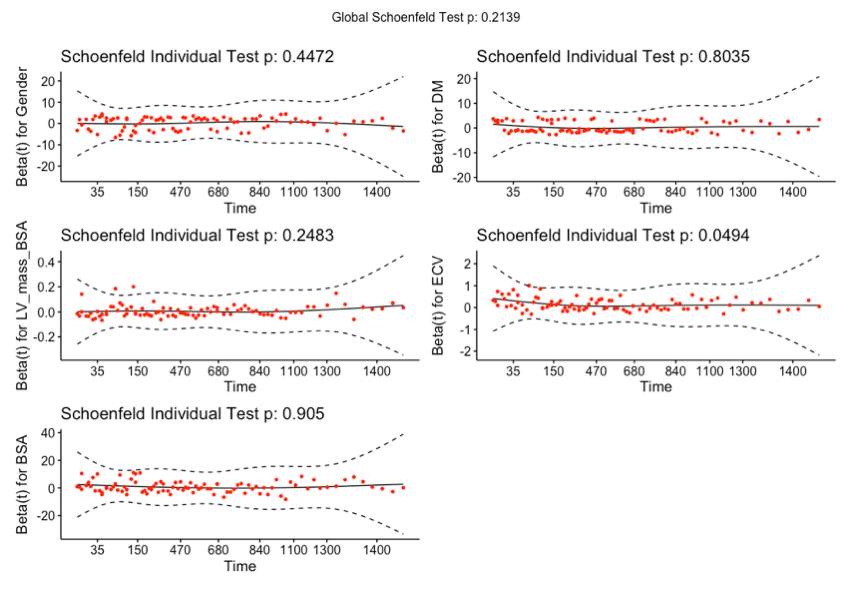
**

**Supplementary Figure 2. Myocardial fibrosis and survival free from all-cause mortality.** Kaplan-Meier curve for survival free from all-cause mortality in patients with chronic obstructive pulmonary disease, according to myocardial fibrosis burden. Myocardial fibrosis was measured using cardiac magnetic resonance imaging extracellular volume (ECV).


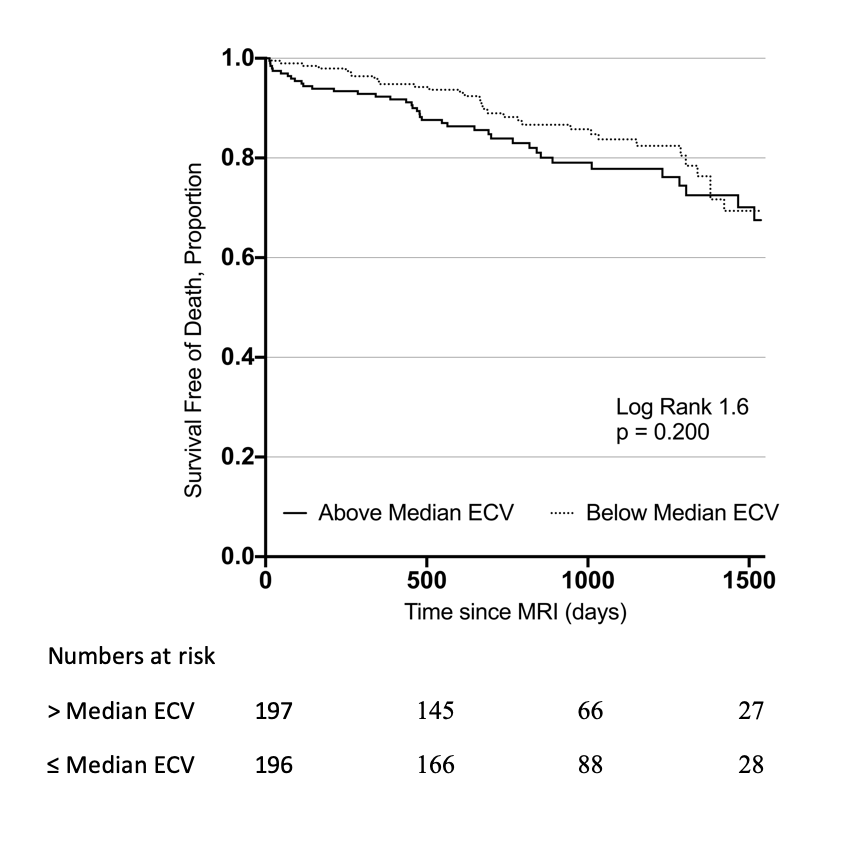


**Supplementary Figure 3. “Dose-response” relationship between extracellular volume (ECV) and outcome**.


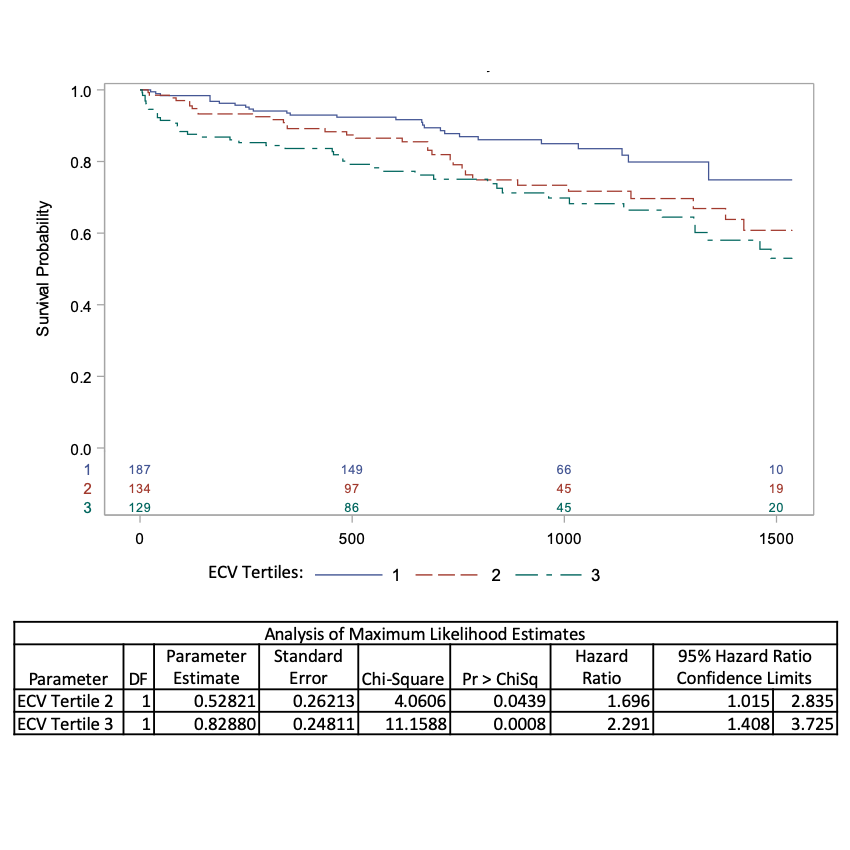


**Supplementary Table 1. Medications**

| **Medication** | **COPD (n=450)** | **Non-COPD (n=122)** | **p value** |
| --- | --- | --- | --- |
| Beta-Blocker | 243 (54%) | 67 (55%) | 0.918 |
| ACEI or ARB | 230 (51%) | 73 (60%) | 0.102 |
| Statin | 227 (50%) | 61 (50%) | 1.000 |

Data presented as number of patients (percentage). ACEI - Angiotensin-converting enzyme inhibitor; ARB - Angiotensin II receptor blocker; COPD – Chronic obstructive pulmonary disease.

**Supplementary Table 2. Parametric mapping values by scanner field strength and site.**

| **Parameter** | **COPD** | **Non-COPD** |
| --- | --- | --- |
| Native T1 |  |  |
| MFT 1.5 T scanner (ms; number of participants) | 1038 (1010 - 1065); n=164 | 1029 (1002 - 1064); n=46 |
| MFT 3 T scanner (ms; number of participants) | 1254 (1217 – 1285); n=188 | 1232 (1200 – 1253); n=54 |
| UPMC 1.5 T scanner (ms; number of participants) | 1014 (981 – 1051); n=87 | 998 (974 – 1023); n=22 |
| Post contrast T1 |  |  |
| T1 MFT 1.5 T scanner (ms; number of participants) | 482 (447 - 528); n=147 | 497 (448 - 527); n=46 |
| T1 MFT 3 T scanner (ms; number of participants) | 529 (476 – 571); n=176 | 501 (465 – 545); n=54 |
| T1 UPMC 1.5 T scanner (ms; number of participants) | 445 (410 – 486); n=87 | 457 (433 – 490); n=22 |
| ECV (%) |  |  |
| MFT 1.5 T scanner (ms; number of participants) | 27.7 (25.5 - 30.7); n=140 | 26.4 (24.3 – 29.5); n=46 |
| MFT 3 T scanner (ms; number of participants) | 27.6 (25.5 – 29.8); n=166 | 25.3 (22.7 – 27.6); n=54 |
| UPMC 1.5 T scanner (ms; number of participants) | 30.2 (27.5 – 33.5); n=87 | 27.7 (25.6 - 29.7); n=22 |

Data presented as median (interquartile range). ECV – Extracellular volume; MFT – Manchester University NHS Foundation Trust; UPMC – University of Pittsburgh Medical Centre. Other abbreviations as per supplementary table 1.

**Supplementary Table 3. Cox regression modelling of all-cause mortality.**

| **Parameter** | **Univariable Model (n = 450)** | | | **Multivariable model (n = 392)^µ^** | | |
| --- | --- | --- | --- | --- | --- | --- |
|  | **χ^2^** | **HR (95% CI)** | **p value** | **χ^2^** | **HR (95% CI)** | **p value** |
| **Demographics** |  |  |  |  |  |  |
| Age (per 1 year increase) | 0.14 | 1.00 (0.98 - 1.03) | 0.713 |  |  |  |
| Gender (male sex) | 4.95 | 1.79 (1.07 - 2.98) | 0.026 | 5.61 | 1.91 (1.12 - 3.25) | 0.018 |
| BSA (per 0.01 m^2^ increase) | 0.55 | 1.00 (1.00 - 1.01) | 0.457 |  |  |  |
| **Comorbidities** |  |  |  |  |  |  |
| Current Smoker | 1.61 | 1.35 (0.85 - 2.15) | 0.205 |  |  |  |
| Ever Smoker | 1.44 | 1.61 (0.74 - 3.50) | 0.231 |  |  |  |
| Diabetes mellitus | 0.86 | 1.26 (0.78 - 2.04) | 0.353 |  |  |  |
| Hypertension | 1.90 | 1.39 (0.87 - 2.20) | 0.168 |  |  |  |
| Dyslipidaemia | 3.08 | 0.67 (0.43 - 1.05) | 0.079 |  |  |  |
| Atrial fibrillation | 2.13 | 1.48 (0.87 - 2.52) | 0.144 | 3.92 | 1.76 (1.01 - 3.08) | 0.048 |
| Coronary revascularisation | 6.61 | 1.84 (1.16 - 2.93) | 0.010 | 7.30 | 2.00 (1.21 - 3.30) | 0.007 |
| **Laboratory and MRI findings** |  |  |  |  |  |  |
| LV EF (per 1% increase)* | 3.70 | 0.99 (0.97 - 1.00) | 0.055 |  |  |  |
| LV mass (per 1 g/m^2^ increase)* | 1.94 | 1.01 (1.00 - 1.02) | 0.163 |  |  |  |
| MI present^†^ | 3.85 | 1.58 (1.00 - 2.50) | 0.050 |  |  |  |
| ECV (per 1% increase)^∆^ | 16.23 | 1.12 (1.00 - 1.18) | <0.001 | 15.94 | 1.13 (1.06 - 1.19) | <0.001 |
| eGFR^Ω^ | 1.05 | 0.99 (0.98 - 1.01) | 0.305 |  |  |  |
| Haematocrit (per 1% increase)^†^ | 10.83 | 0.94 (0.91 - 0.98) | 0.001 |  |  |  |

Data presented as mean ± standard deviation or median (interquartile range) depending on distribution. *n = 435; ^†^n=425; ^∆^n=393; ^Ω^n=449, per 1 ml/min per 1.73 m2 increase; ^µ^Stepwise model performed in 392 patients without missing data. BSA – Body surface area; CI – Confidence interval; EF – Ejection fraction; eGFR – Estimated glomerular filtration rate; HR – Hazard ratio; LV – Left ventricle; MI – Myocardial infarction; MRI – Magnetic resonance imaging. Other abbreviations as per supplementary tables 1-2.

**Supplementary Table 4. Cox regression modelling of the combined endpoint of hospitalisation for heart failure or all-cause mortality including native myocardial T1 mapping instead of ECV as a variable**

| **Parameter** | **Univariable Model (n = 450)** | | | **Multivariable model (n = 402)^µ^** | | |
| --- | --- | --- | --- | --- | --- | --- |
|  | **χ^2^** | **HR (95% CI)** | **p value** | **χ^2^** | **HR (95% CI)** | **p value** |
| **Demographics** |  |  |  |  |  |  |
| Age (per 1 year increase) | 0.46 | 1.01 (0.99 - 1.03) | 0.499 |  |  |  |
| Gender (male sex) | 1.59 | 1.31 (0.86 - 2.00) | 0.207 |  |  |  |
| BSA (per 0.01 m^2^ increase) | 1.12 | 1.00 (1.00 - 1.01) | 0.289 |  |  |  |
| **Comorbidities** |  |  |  |  |  |  |
| Current Smoker | 0.72 | 1.20 (0.79 - 1.80) | 0.395 |  |  |  |
| Ever Smoker | 0.76 | 1.32 (0.71 - 2.47) | 0.384 |  |  |  |
| Diabetes mellitus | 3.74 | 1.50 (1.00 - 2.27) | 0.053 |  |  |  |
| Hypertension | 3.06 | 1.44 (0.96 - 2.15) | 0.080 |  |  |  |
| Dyslipidaemia | 2.94 | 0.71 (0.48 - 1.05) | 0.086 |  |  |  |
| Atrial fibrillation | 1.41 | 1.33 (0.83 - 2.14) | 0.236 |  |  |  |
| Coronary revascularisation | 3.52 | 1.49 (0.98 - 2.27) | 0.061 | 5.62 | 1.74 (1.10 - 2.74) | 0.018 |
| **Laboratory and MRI findings** |  |  |  |  |  |  |
| LV EF (per 1% increase)* | 10.26 | 0.98 (0.97 - 0.99) | 0.001 | 12.54 | 0.98 (0.96 - 0.99) | <0.001 |
| LV mass (per 1 g/m^2^ increase)* | 6.71 | 1.01 (1.00 - 1.02) | 0.010 |  |  |  |
| MI present^†^ | 5.27 | 1.60 (1.07 - 2.39) | 0.022 |  |  |  |
| Native T1 (per 1 ms increase)^∆^ | 1.58 | 1.00 (1.00 – 1.00) | 0.209 |  |  |  |
| eGFR^Ω^ | 3.65 | 0.99 (0.98 - 1.00) | 0.056 |  |  |  |
| Haematocrit (per 1% increase)^†^ | 18.42 | 0.93 (0.90 - 0.96) | <0.001 | 8.38 | 0.95 (0.92 - 0.98) | 0.004 |

Data presented as mean ± standard deviation or median (interquartile range) depending on distribution. *n = 435; ^†^n=425; .∆ n=439; ^Ω^n=449, per 1 ml/min per 1.73 m2 increase; ^µ^Stepwise model performed in 402 patients without missing data. Abbreviations as per supplementary tables 1-3.

**Supplementary Table 5. Cox regression modelling of the combined endpoint of hospitalisation for heart failure or all-cause mortality including post-contrast myocardial T1 mapping instead of ECV as a variable**

| **Parameter** | **Univariable Model (n = 450)** | | | **Multivariable model (n = 392)^µ^** | | |
| --- | --- | --- | --- | --- | --- | --- |
|  | **χ^2^** | **HR (95% CI)** | **p value** | **χ^2^** | **HR (95% CI)** | **p value** |
| **Demographics** |  |  |  |  |  |  |
| Age (per 1 year increase) | 0.46 | 1.01 (0.99 - 1.03) | 0.499 |  |  |  |
| Gender (male sex) | 1.59 | 1.31 (0.86 - 2.00) | 0.207 |  |  |  |
| BSA (per 0.01 m^2^ increase) | 1.12 | 1.00 (1.00 - 1.01) | 0.289 |  |  |  |
| **Comorbidities** |  |  |  |  |  |  |
| Current Smoker | 0.72 | 1.20 (0.79 - 1.80) | 0.395 |  |  |  |
| Ever Smoker | 0.76 | 1.32 (0.71 - 2.47) | 0.384 |  |  |  |
| Diabetes mellitus | 3.74 | 1.50 (1.00 - 2.27) | 0.053 |  |  |  |
| Hypertension | 3.06 | 1.44 (0.96 - 2.15) | 0.080 |  |  |  |
| Dyslipidaemia | 2.94 | 0.71 (0.48 - 1.05) | 0.086 |  |  |  |
| Atrial fibrillation | 1.41 | 1.33 (0.83 - 2.14) | 0.236 |  |  |  |
| Coronary revascularisation | 3.52 | 1.49 (0.98 - 2.27) | 0.061 | 5.85 | 1.76 (1.11 - 2.79) | 0.016 |
| **Laboratory and MRI findings** |  |  |  |  |  |  |
| LV EF (per 1% increase)* | 10.26 | 0.98 (0.97 - 0.99) | 0.001 | 12.27 | 0.98 (0.96 - 0.99) | <0.001 |
| LV mass (per 1 g/m^2^ increase)* | 6.71 | 1.01 (1.00 - 1.02) | 0.010 |  |  |  |
| MI present^†^ | 5.27 | 1.60 (1.07 - 2.39) | 0.022 |  |  |  |
| Post contrast T1 (per 1 ms increase)^∆^ | 1.72 | 1.00 (1.00 – 1.01) | 0.190 |  |  |  |
| eGFR^Ω^ | 3.65 | 0.99 (0.98 - 1.00) | 0.056 |  |  |  |
| Haematocrit (per 1% increase)^†^ | 18.42 | 0.93 (0.90 - 0.96) | <0.001 | 8.24 | 0.95 (0.92 - 0.98) | 0.004 |

Data presented as mean ± standard deviation or median (interquartile range) depending on distribution. *n = 435; ^†^n=425; .∆ n=410; ^Ω^n=449, per 1 ml/min per 1.73 m2 increase; ^µ^Stepwise model performed in 392 patients without missing data. Abbreviations as per supplementary tables 1-4.

**Supplementary Table 6. Cox regression modelling of the combined endpoint of hospitalisation for heart failure or all-cause mortality including FEV1 as a variable.**

| **Parameter** | **Univariable Model (n = 450)** | | | **Multivariable model (n = 192)^µ^** | | |
| --- | --- | --- | --- | --- | --- | --- |
|  | **χ^2^** | **HR (95% CI)** | **p value** | **χ^2^** | **HR (95% CI)** | **p value** |
| **Demographics** |  |  |  |  |  |  |
| Age (per 1 year increase) | 0.46 | 1.01 (0.99 - 1.03) | 0.499 |  |  |  |
| Gender (male sex) | 1.59 | 1.31 (0.86 - 2.00) | 0.207 |  |  |  |
| BSA (per 0.01 m^2^ increase) | 1.12 | 1.00 (1.00 - 1.01) | 0.289 |  |  |  |
| **Comorbidities** |  |  |  |  |  |  |
| Current Smoker | 0.72 | 1.20 (0.79 - 1.80) | 0.395 |  |  |  |
| Ever Smoker | 0.76 | 1.32 (0.71 - 2.47) | 0.384 |  |  |  |
| Diabetes mellitus | 3.74 | 1.50 (1.00 - 2.27) | 0.053 |  |  |  |
| Hypertension | 3.06 | 1.44 (0.96 - 2.15) | 0.080 |  |  |  |
| Dyslipidaemia | 2.94 | 0.71 (0.48 - 1.05) | 0.086 |  |  |  |
| Atrial fibrillation | 1.41 | 1.33 (0.83 - 2.14) | 0.236 |  |  |  |
| Coronary revascularisation | 3.52 | 1.49 (0.98 - 2.27) | 0.061 |  |  |  |
| **Laboratory and MRI findings** |  |  |  |  |  |  |
| LV EF (per 1% increase)* | 10.26 | 0.98 (0.97 - 0.99) | 0.001 |  |  |  |
| LV mass (per 1 g/m^2^ increase)* | 6.71 | 1.01 (1.00 - 1.02) | 0.010 |  |  |  |
| MI present^†^ | 5.27 | 1.60 (1.07 - 2.39) | 0.022 |  |  |  |
| ECV (per 1% increase)^∆^ | 30.45 | 1.15 (1.09 - 1.21) | <0.001 | 12.04 | 1.15 (1.06 - 1.25) | 0.001 |
| eGFR^Ω^ | 3.65 | 0.99 (0.98 - 1.00) | 0.056 |  |  |  |
| Haematocrit (per 1% increase)^†^ | 18.42 | 0.93 (0.90 - 0.96) | <0.001 |  |  |  |
| **Lung function tests** |  |  |  |  |  |  |
| FEV_1_ (L/s)^∂^ | 2.54 | 0.66 (0.39 - 1.10) | 0.111 |  |  |  |

Data presented as mean ± standard deviation or median (interquartile range) depending on distribution. *n=435; ^†^n=425; ^∆^n=393; ^Ω^n=449, per 1 ml/min per 1.73 m2 increase; ^∂^n=235; ^µ^Stepwise model performed in 192 patients without missing data. FEV_1_ - Forced expiratory volume in one second. Other abbreviations as per Supplementary table 1-7.

**Supplementary Table 7. Cox regression modelling of the combined endpoint of hospitalisation for heart failure or all-cause mortality including right ventricular ejection fraction as a variable.**

| **Parameter** | **Univariable Model (n = 450)** | | | **Multivariable model (n = 305)^µ^** | | |
| --- | --- | --- | --- | --- | --- | --- |
|  | **χ^2^** | **HR (95% CI)** | **p value** | **χ^2^** | **HR (95% CI)** | **p value** |
| **Demographics** |  |  |  |  |  |  |
| Age (per 1 year increase) | 0.46 | 1.01 (0.99 - 1.03) | 0.499 |  |  |  |
| Gender (male sex) | 1.59 | 1.31 (0.86 - 2.00) | 0.207 |  |  |  |
| BSA (per 0.01 m^2^ increase) | 1.12 | 1.00 (1.00 - 1.01) | 0.289 |  |  |  |
| **Comorbidities** |  |  |  |  |  |  |
| Current Smoker | 0.72 | 1.20 (0.79 - 1.80) | 0.395 |  |  |  |
| Ever Smoker | 0.76 | 1.32 (0.71 - 2.47) | 0.384 |  |  |  |
| Diabetes mellitus | 3.74 | 1.50 (1.00 - 2.27) | 0.053 |  |  |  |
| Hypertension | 3.06 | 1.44 (0.96 - 2.15) | 0.080 |  |  |  |
| Dyslipidaemia | 2.94 | 0.71 (0.48 - 1.05) | 0.086 |  |  |  |
| Atrial fibrillation | 1.41 | 1.33 (0.83 - 2.14) | 0.236 |  |  |  |
| Coronary revascularisation | 3.52 | 1.49 (0.98 - 2.27) | 0.061 | 4.14 | 1.95 (1.02 - 3.70) | 0.042 |
| **Laboratory and MRI findings** |  |  |  |  |  |  |
| LV EF (per 1% increase)* | 10.26 | 0.98 (0.97 - 0.99) | 0.001 |  |  |  |
| RV EF (per 1% increase)^II^ | 0.49 | 0.99 (0.97 – 1.01) | 0.486 |  |  |  |
| LV mass (per 1 g/m^2^ increase)* | 6.71 | 1.01 (1.00 - 1.02) | 0.010 |  |  |  |
| MI present^†^ | 5.27 | 1.60 (1.07 - 2.39) | 0.022 |  |  |  |
| ECV (per 1% increase)^∆^ | 30.45 | 1.15 (1.09 - 1.21) | <0.001 | 14.41 | 1.15 (1.07 - 1.23) | <0.001 |
| eGFR^Ω^ | 3.65 | 0.99 (0.98 - 1.00) | 0.056 |  |  |  |
| Haematocrit (per 1% increase)^†^ | 18.42 | 0.93 (0.90 - 0.96) | <0.001 |  |  |  |

Data presented as mean ± standard deviation or median (interquartile range) depending on distribution. *n=435; ^†^n=425; ^∆^n=393; ^Ω^n=449, per 1 ml/min per 1.73 m2 increase; ^II^n=348. ^µ^Stepwise model performed in 305 patients without missing data. RV – Right ventricle. Other abbreviations as per supplementary table 1-8.

**Supplementary Table 8. Characteristics of participants with acutely exacerbating chronic obstructive pulmonary disease and current smokers**

| **Parameter** | **Healthy volunteers (n=15)** | **Smokers (n=15)** | **p  value^¥^** | **Acute COPD  (n=15)** | **p  value^¥^** |
| --- | --- | --- | --- | --- | --- |
| **Demographics** |  |  |  |  |  |
| Age (years) | 53 (38 - 60) | 53 (44 - 58) | 0.663 | 68 (58 - 73)* | 0.002 |
| Gender (Female) | 7 (47%) | 5 (33%) | 0.710 | 9 (60%)* | 0.715 |
| **Comorbidities** |  |  |  |  |  |
| COPD | 0 (0%) | 0 (0%) |  | 15 (100%) |  |
| Depression | 0 (0%) | 3 (20%) |  | 4 (27%) |  |
| Dyslipidaemia | 0 (0%) | 2 (13%) |  | 2 (13%) |  |
| Hypertension | 0 (0%) | 1 (7%) |  | 3 (20%) |  |
| Diabetes Mellitus | 0 (0%) | 1 (7%) |  | 1 (7%) |  |
| Gallstones | 0 (0%) | 1 (7%) |  | 1 (7%) |  |
| Hypothyroidism | 0 (0%) | 1 (7%) |  | 1 (7%) |  |
| Gastritis / GORD | 0 (0%) | 1 (7%) |  | 1 (7%) |  |
| **Smoking history** |  |  |  |  |  |
| Current smoker | 0 (0%) | 15 (100% | <0.001 | 7 (47%) | 0.006 |
| Ever smoker | 0 (0%) | 15 (100%) | <0.001 | 15 (100%) | <0.001 |
| Smoking pack-years | 0 (0 - 0) | 29 (16 - 48) | <0.001 | 50 (29 - 59) | <0.001 |

Data presented as mean ± standard deviation or median (interquartile range) depending on distribution. ^¥^vs. controls. COPD – Chronic obstructive pulmonary disease; GORD – Gastro-oesophageal reflux disease. *Of the 12 participants with COPD who completed the acute evaluation, median age was 63 (56 – 75) years and 7 (58%) were female.

**Supplementary Table 9. Measurements of inflammation in acute and stable chronic obstructive pulmonary disease and current smokers.**

| **Parameter** | **Controls (n=15)** | **Smokers (n=15)** | **p  value*** | **Acute COPD  (n=15)** | **p  value*** | **Stable COPD  (n=10)** | **p  value*** | **p  value**^†^ |
| --- | --- | --- | --- | --- | --- | --- | --- | --- |
| **Laboratory findings** |  |  |  |  |  |  |  |  |
| WBC (x10^9^/L) | 5.9 (4.9 - 6.1) | 8.5 (7.3 - 9.4) | 0.001 | 9.6 (8.0 – 11.0) | 0.001 | 8.5 (7.0 - 10.8) | 0.002 | 0.123 |
| CRP (mg/L) | 0 (0 - 2) | 2 (0 - 4) | 0.123 | 6 (3 - 11) | 0.001 | 4 (0 - 7) | 0.012 | 0.017 |
| **MRI findings** |  |  |  |  |  |  |  |  |
| USPIO MRI R2*/R1 | 24.9 ± 2.6^‡^ | 27.1 ± 3.0 | 0.123 | 30.2 ± 3.2^§^ | 0.005 | 31.0 ± 5.6 | 0.025 | 0.537 |
| T1 (ms) | 1006 ± 30 | 1007 ± 24 | 0.939 | 1041 ± 33^ll^ | 0.007 | 1018 ± 34 | 0.358 | 0.203 |
| T2 (ms) | 50 ± 2 | 50 ± 2 | 0.752 | 51 ± 3^ll^ | 0.178 | 51 ± 2 | 0.130 | 0.755 |
| K^trans^ (min^-1^) | 0.32 ± 0.08^§^ | 0.35 ± 0.07 | 0.323 | 0.39 ± 0.14^ll^ | 0.155 | 0.32 ± 0.07^§^ | 0.997 | 0.191 |

Data presented as mean ± standard deviation or median (interquartile range) depending on distribution. *vs. controls; ^†^acute vs. stable COPD; ^‡^n=6; ^§^n=9, ^ll^n=12. CRP – C reactive protein; K^trans^ – Transfer constant; R1 - longitudinal relaxation rate (1/T1 relaxation time); R2* - transverse relaxation rate in the presence of static field inhomogeneities (1/T2* relaxation time); USPIO – Ultrasmall superparamagnetic particles of iron oxide; WBC – white blood cell count. Other abbreviations as per supplementary tables 1 – 8.

**Supplementary Table 10. R2* change over time.**

| **R2*** | **Healthy volunteers (n=15)** | **Smokers (n=15)** | **p  value^∆^** | **Acute COPD  (n=12)** | **p  value^∆^** | **Stable COPD  (n=10)** | **p  value*** | **p  value^†^** |
| --- | --- | --- | --- | --- | --- | --- | --- | --- |
| Scan 1 (s^-1^) | 27.6 ± 3.6 | 28.4 ± 3.3 | 0.015 | 30.4 ± 6.8 | 0.038 | 30.3 ± 4.7 | 0.005 | 0.356 |
| Scan 2 (s^-1^) | 40.3 ± 8.6 | 47.2 ± 8.5 |  | 53.1 ± 9.4^µ^ |  | 54.3 ± 7.1 |  |  |
| Scan 3 (s^-1^) | 29.0 ± 4.4 | 33.4 ± 4.1 |  | 37.4 ± 4.6^∂^ |  | 39.5 ± 7.8 |  |  |

Change in R2* over time compared using generalised estimating equations. ^∆^vs. controls; ^†^acute vs. stable COPD; ^µ^n=11; ^∂^n=9. COPD - chronic obstructive pulmonary disease.
